# Chlamydia muridarum Genital and Gastrointestinal Infection Tropism Is Mediated by Distinct Chromosomal Factors

**DOI:** 10.1128/IAI.00141-18

**Published:** 2018-06-21

**Authors:** Sandra G. Morrison, Amanda M. Giebel, Evelyn C. Toh, Horace J. Spencer, David E. Nelson, Richard P. Morrison

**Affiliations:** a Department of Microbiology & Immunology, University of Arkansas for Medical Sciences, Little Rock, Arkansas, USA; b Department of Biostatistics, University of Arkansas for Medical Sciences, Little Rock, Arkansas, USA; c Department of Microbiology & Immunology, Indiana University School of Medicine, Indianapolis, Indiana, USA; Washington State University

**Keywords:** *Chlamydia*, gastrointestinal infection, genital tract immunity, intracellular bacteria, intracellular pathogen, sexually transmitted diseases

## Abstract

Some members of the genus Chlamydia, including the human pathogen Chlamydia trachomatis, infect multiple tissues, including the genital and gastrointestinal (GI) tracts. However, it is unknown if bacterial targeting to these sites is mediated by multifunctional or distinct chlamydial factors. We previously showed that disruption of individual large clostridial toxin homologs encoded within the Chlamydia muridarum plasticity zone were not critical for murine genital tract infection. Here, we assessed whether cytotoxin genes contribute to C. muridarum GI tropism. Infectivity and shedding of wild-type (WT) C. muridarum and three mutants containing nonsense mutations in different cytotoxin genes, *tc0437*, *tc0438*, and *tc0439*, were compared in mouse genital and GI infection models. One mutant, which had a nonsense mutation in *tc0439*, was highly attenuated for GI infection and had a GI 50% infectious dose (ID_50_) that was 1,000 times greater than that of the WT. GI inoculation with this mutant failed to elicit anti-chlamydial antibodies or to protect against subsequent genital tract infection. Genome sequencing of the *tc0439* mutant revealed additional chromosomal mutations, and phenotyping of additional mutants suggested that the GI attenuation might be linked to a nonsense mutation in *tc0600*. The molecular mechanism underlying this dramatic difference in tissue-tropic virulence is not fully understood. However, isolation of these mutants demonstrates that distinct chlamydial chromosomal factors mediate chlamydial tissue tropism and provides a basis for vaccine initiatives to isolate chlamydia strains that are attenuated for genital infection but retain the ability to colonize the GI tract and elicit protective immune responses.

## INTRODUCTION

Chlamydia trachomatis infection is the most common bacterial sexually transmitted infection in the United States, with nearly 1.6 million cases reported to the U.S. Centers for Disease Control and Prevention (CDC) in 2016 (1). Many C. trachomatis infections are asymptomatic, so the actual number of cases is likely to be much higher (2–4). When chlamydia infection is diagnosed, it is effectively treated with antibiotics; however, undetected genital infections in women can persist and ascend, to cause infection and subsequent inflammation in the upper genital tract (5). Upper genital tract inflammation may cause pelvic inflammatory disease, salpingitis, tubal scarring, ectopic pregnancy, and infertility. In addition to the urogenital tract, C. trachomatis infects the conjunctiva, pharynx, respiratory tract, rectum, and gastrointestinal (GI) tract. The recent recognition that rectal infections are frequent in women who do not report traditional rectal chlamydia infection risk factors (6), and the premise that the GI tract may serve as a site for persisting infection and as a reservoir for urogenital reinfection (7–10), has renewed interest in understanding the molecular basis of chlamydial GI colonization and pathogenesis.

The chlamydial plasticity zone (PZ) is a region of high genetic diversity in the genomes of otherwise highly conserved Chlamydia spp. (11, 12). Some PZ genes mediate chlamydial immune evasion, but the functions of most of these genes are unknown (13–16). Using a murine genital tract infection model, we previously assessed the virulence of Chlamydia muridarum mutants containing nonsense mutations in various PZ genes, including three cytotoxin genes (*tc0437*, *tc0438*, and *tc0439*) (17). These mutants elicited genital infections similar to those caused by their parent, as assessed by bacterial shedding and duration of infection, although infection with the mutants generally resulted in fewer occurrences of hydrosalpinx (17). However, because the chlamydial cytotoxin genes share homology with large clostridial toxin genes (18), which are key Clostridium difficile GI virulence factors (19), we sought to determine if the C. muridarum cytotoxin mutants showed altered virulence for GI infection.

In the current study, the pathogenicities of the three cytotoxin nonsense mutants were compared in two GI infection models by assessing bacterial shedding, anti-chlamydia antibody responses, and immunity to genital infection. We found that nonsense mutations in individual cytotoxin genes *per se* did not alter the virulence of C. muridarum infection for either genital or GI infections. However, the profound attenuation of one of the cytotoxin mutants for GI, but not genital, infection was associated with an additional background nonsense mutation in the conserved chlamydial hypothetical protein gene *tc0600*, suggesting that this gene might be a GI-specific virulence factor. Overall, our results show that distinct chromosomal genes mediate C. muridarum genital and GI infection and suggest that it might be possible to construct vaccine strains that are attenuated for genital tract infection but are able to colonize the GI tract to elicit transmucosal genital tract protection.

## RESULTS

### C. muridarum cytotoxin mutants are virulent in the murine genital tract.

**FIG 1:**
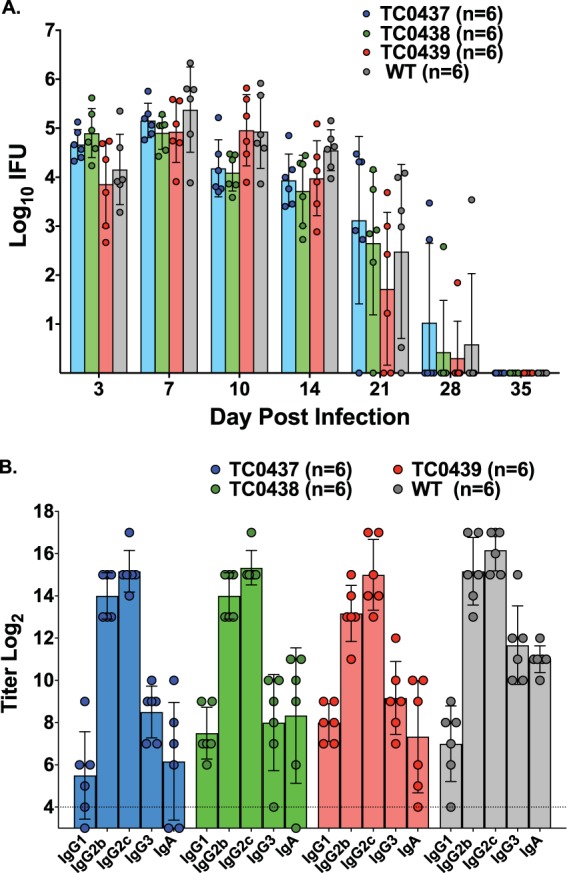
C. muridarum toxin mutants are virulent in the genital tract. C57BL/6 mice were pretreated with medroxyprogesterone acetate and challenged with 5 × 10^4^ IFUs of the WT (*n* = 6) or toxin mutants (*n* = 6 for each mutant). (A) Vaginal vaults were swabbed at the indicated times, and IFUs were enumerated on HeLa cell monolayers by immunofluorescence. Data are presented as the geometric mean ± standard deviation (SD) of IFUs recovered at the indicated days postinfection. Statistical analysis revealed differences in bacterial shedding over time among the strains (group-by-day *P* value, 0.0049). Comparison of the WT profile to each of the mutant profiles revealed that the WT was statistically different from TC0437 and TC0438 but not TC0439. The differences between TC0437 and the WT approached statistical significance at day 10 (*P* < 0.065) and day 14 (*P* < 0.094), and TC0438 was significantly different from WT at day 10 (*P* < 0.016). No other differences were detected. (B) Sera collected 49 days following genital infection were analyzed by EB ELISA. Antibody responses are presented as mean log_2_ titers ± SD for each of the indicated Ig classes. A dashed line indicates cutoff values for a positive serologic response. The antibody responses were remarkably similar, with the following exceptions: WT versus TC0437 and WT versus TC0439 IgA titers, *P* < 0.01; WT versus TC0437 and WT versus TC0438 IgG3 titers, *P* < 0.05. No other differences were detected.

The goal of the current study was to determine if C. muridarum cytotoxin mutants have tissue-specific virulence defects. Therefore, it was first important to verify the virulence of all strains in the genital infection model (17) and to measure baseline immune responses for comparison to GI infections. Mice were intravaginally inoculated with the wild-type parent strain (WT) or with mutants (TC0437, TC0438, or TC0439) having nonsense mutations in one of the three cytotoxin genes (*tc0437*, *tc0438*, or *tc0439*, respectively), and infection was monitored by enumerating chlamydiae collected from cervicovaginal swabs. The magnitude and duration of chlamydial shedding between WT- and mutant-inoculated animals were similar, with the only significant difference detected between WT and TC0438 at day 10 (*P* < 0.016) (Fig. 1A). Furthermore, the overall immunoglobulin class- and subclass-specific anti-chlamydia antibody responses elicited by infection with the mutants was not markedly different from those of the WT, but slight differences were observed in the titers of some responses (Fig. 1B). Thus, measures of chlamydial shedding, infection duration, and immunogenicity confirmed that disruption of individual cytotoxin genes did not overtly impact the virulence of C. muridarum in the mouse genital tract.

### C. muridarum mutant TC0439 displays unique tissue-specific virulence.

**FIG 2:**
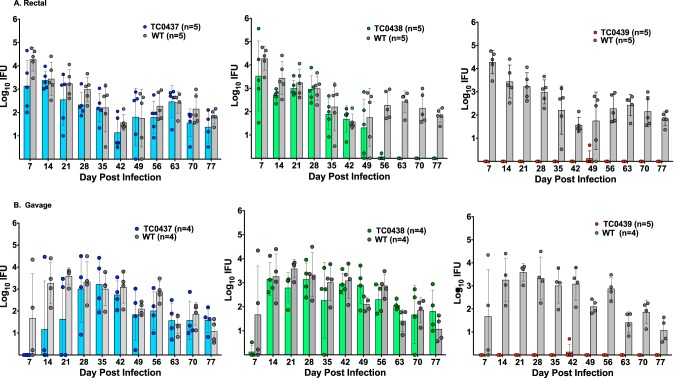
TC0439 is highly attenuated for GI infection. C57BL/6 mice were challenged by rectal (A) or gavage (B) inoculation with the WT or mutants to measure virulence for GI infection. For rectal inoculation, 10 μl containing 1 × 10^5^ IFUs was placed intrarectally. Gavage inoculation was accomplished by inoculation of 100 μl containing 1 × 10^6^ IFUs via a feeding tube. Infection was assessed weekly by swabbing the rectum and counting IFUs on HeLa cell monolayers. Data are presented as the geometric mean ± SD of IFUs at the indicated time points. The number of mice per group is indicated in the figure. For comparison, the infection curve for WT rectal (A) and gavage (B) infection is replicated in each panel. (A) Rectal challenge. The test results of the overall interaction effect were found to be statistically significant (*P* < 0.0001). The interaction between TC0437 and the WT was not significantly different (*P* > 0.63), and the test of the main strain effect was not statistically significant (*P* > 0.063). The interaction effect for TC0438 and the WT was statistically significant (*P* < 0.0001). A comparison performed for each day found significant differences from the WT at day 56 and beyond (*P* < 0.008). Differences at other time points were not significant. TC0439 was significantly different from the WT at all time points (*P* < 0.001). (B) Gavage challenge. TC0437 was not found to be significantly different from the WT (interaction, *P* > 0.085; main strain effect, *P* > 0.11); TC0438 was not found to be significantly different from the WT (interaction, *P* > 0.19; main strain effect, *P* > 0.35); and TC0439 was found to be significantly different from the WT (interaction, *P* < 0.020; main strain effect, *P* < 0.0001).

Chlamydiae have been isolated from the GI tracts of both animals and humans (7). In mice, C. muridarum establishes long-lasting GI infections characterized by minimal tissue pathology (10, 20–23). Because C. muridarum cytotoxin genes are homologous to genes that are critical for the colonization of the GI tract by other bacterial pathogens (24, 25), we sought to determine if the GI tropism of chlamydial cytotoxin mutants is altered. GI infections were established by intrarectal (Fig. 2A) or oral gavage (Fig. 2B) inoculation. The WT produced long-lasting infections by both inoculation routes. GI shedding from WT-infected mice measured between 10^3^ and 10^4^ inclusion-forming units (IFUs) during early infection (weeks 1 to 4) and then decreased to 10^1^ to 10^3^ IFUs later in infection (weeks 5 to 11). The course of GI infections produced by either inoculation route with mutants TC0437 and TC0438 was remarkably similar to that of the WT, although mice inoculated intrarectally with TC0438 did resolve GI infection by 8 weeks postinoculation (*P* < 0.008). In contrast, the attenuation of TC0439 in both GI infection models was striking (Fig. 2). Neither intrarectal nor oral gavage inoculation with TC0439 resulted in productive infection (*P* < 0.001). Thus, TC0439 displayed a unique tissue-restricted virulence phenotype, retaining full virulence for genital tract infection (Fig. 1) while exhibiting highly attenuated GI infection (Fig. 2).

### Immunological studies corroborate the attenuation of TC0439 for GI infection.

**FIG 3:**
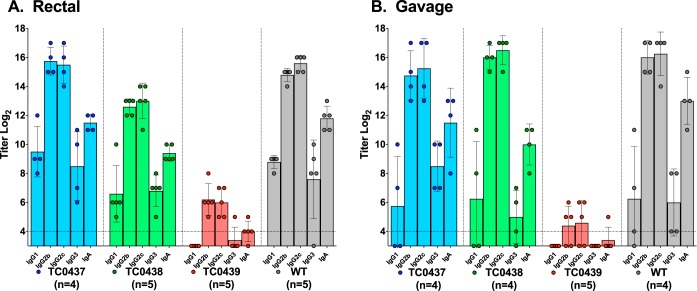
TC0439 fails to elicit robust antibody responses following GI infection. As an additional assessment of infection, the antibody responses of GI infected mice (Fig. 2) were measured. Sera collected 81 days following rectal (A) or gavage (B) challenge were analyzed by EB ELISA. The number of mice per group is indicated on the figure. Antibody responses are presented as the mean log_2_ titers ± SD for each of the indicated Ig classes. Dashed lines indicate cutoff values for positive serologic responses. The antibody responses of mice infected following rectal or gavage inoculation with mutant TC0439 were negative or just above background responses and were significantly lower than responses elicited by WT infection (*P* < 0.016 for all Ig classes tested, except IgG3, which was not significant, *P* > 0.05). Antibody responses elicited by TC0437 and TC0438 were not significantly different from that of the WT (*P* > 0.05), except for TC0438 rectal challenge (IgG2b, IgG2c, and IgA, *P* < 0.008).

We inferred, from the chlamydial shedding data collected using rectal swabs, that TC0439 was attenuated for GI infection. An alternative explanation for that observation is that TC0439 established infection in the upper GI tract or disseminated to distant tissues and rectal culture failed to detect those infections. We differentiated those possibilities by measuring the anti-chlamydial antibody responses and the ability of GI-challenged mice to defend against genital reinfection. Infection of the murine GI tract with the WT by intrarectal (Fig. 3A) or oral gavage (Fig. 3B) inoculation elicited robust anti-chlamydia antibody responses. Similar antibody responses developed following infection with TC0437 and TC0438. In contrast, the antibody responses elicited following intrarectal (Fig. 3A) or oral gavage (Fig. 3B) inoculation with TC0439 were below or slightly above baseline levels of uninfected mice (*P* < 0.02).

**FIG 4:**
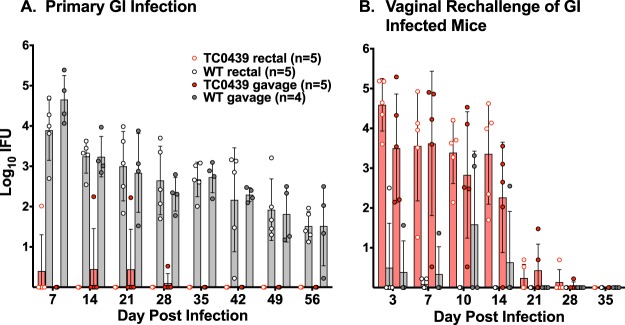
GI infection with TC0439 does not protect against genital challenge. (A) Mice were infected by either gavage or intrarectal inoculation with the WT or TC0439, and GI infection was monitored by enumerating IFUs from rectal swabs. (B) At 118 days following GI infection, mice were treated with doxycycline for 10 days, rested 2 weeks, treated with medroxyprogesterone acetate and then rechallenged vaginally with the homologous strain. Data are presented as the geometric mean ± SD. The number of mice per group is indicated on the figure. As shown previously (Fig. 1), mutant TC0439 was highly attenuated for GI infection (A) (TC0439 compared to the WT by both gavage and rectal inoculation, *P* < 0.0001). Gastrointestinal infection (by gavage or intrarectal inoculation) with the WT conferred striking protective immunity (decreased bacterial shedding and shorter duration of infection) to vaginal challenge, whereas neither rectal nor gavage inoculation of TC0439 protected against vaginal challenge (*P* < 0.0005) (B). All mice receiving GI (rectal or gavage inoculation) WT infection were protected from the development of hydrosalpinx upon vaginal challenge.

**FIG 5:**
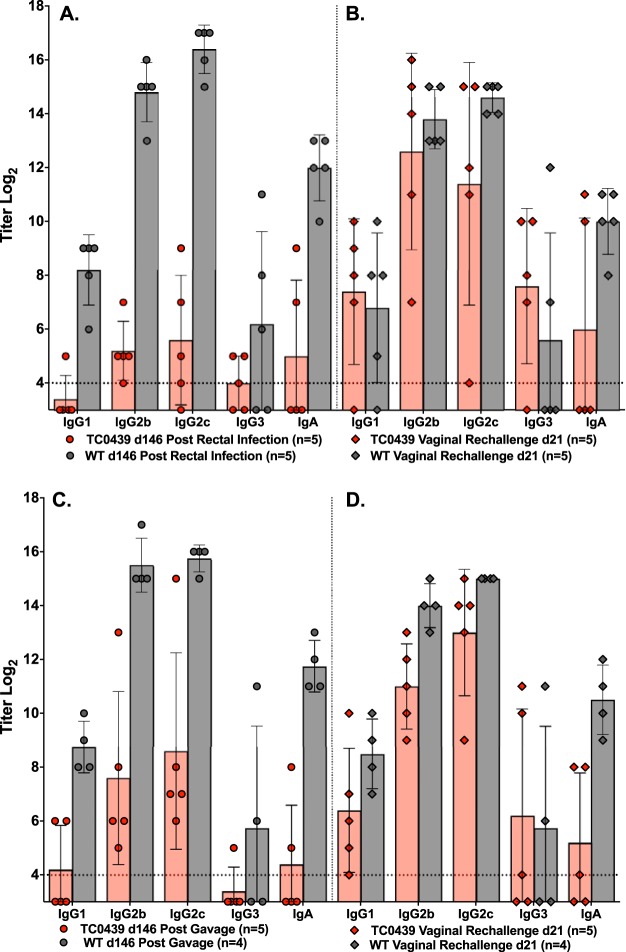
Antibody response of TC0439 GI-infected mice increases following vaginal rechallenge. Sera were collected from the WT- and TC0439-infected mice described in the legend to Fig. 4 and analyzed by EB ELISA. Antibody responses are presented as mean log_2_ titers ± SD for each of the indicated Ig classes. Dashed lines indicate cutoff values for positive serologic responses. (A and C) Anti-chlamydia antibody responses of mice challenged intrarectally (A) or by gavage (C) at 146 days following GI challenge. (B and D) Sera were collected 21 days following vaginal rechallenge of mice infected rectally (B) or by gavage (D) and analyzed by EB ELISA. WT GI infection (both rectal and gavage infection) elicited robust anti-chlamydia antibody responses, and titers did not significantly change upon vaginal rechallenge (*P* > 0.05). TC0439 elicited significantly lower anti-chlamydial antibody responses than the WT (*P* < 0.032 for IgG1, IgG2b, IgG2c, and IgA but not for IgG3) following primary infection either rectally or by gavage (A and C), and antibody responses following vaginal challenge rose to levels comparable to that of the WT (rectal challenge in panel B, *P* > 0.05 for all Ig class and subclass responses; gavage challenge in panel D, *P* < 0.032 for IgG2b, IgG2c, and IgA, and *P* > 0.05 for IgG1 and IgG3).

The inability of TC0439 to productively infect the GI tract and induce anti-chlamydial antibodies suggested that its GI virulence was severely impaired. For an independent virulence measure, we assessed whether GI infection with TC0439 protected against subsequent genital tract challenge (23). Mice were challenged by either intrarectal or oral gavage inoculation with the WT or mutant TC0439, and GI infection was monitored by rectal swab for 11 weeks (Fig. 4A). Approximately 17 weeks postinoculation, mice were treated with doxycycline to cure residual infection, treated with medroxyprogesterone acetate, and challenged vaginally with the corresponding chlamydial strain. Mice infected with the WT, by either intrarectal or gavage inoculation, were markedly protected against WT genital tract infection (*P* < 0.0005) (Fig. 4B). In contrast, GI inoculation of mice with TC0439 did not protect against subsequent TC0439 genital infection. The course of genital infection (chlamydial shedding and infection duration) in mice receiving a prior GI challenge with TC0439 was remarkably similar to that of primary genital tract infection with this mutant (Fig. 1A). Moreover, the marginal anti-chlamydia antibody response elicited by TC0439 following GI infection was increased to a level typically observed following primary vaginal challenge (Fig. 5A and B). Also, 4 days prior to vaginal challenge (143 days post-GI infection), all GI WT-challenged mice (5 of 5 intrarectally infected and 4 of 4 infected by gavage) were vaginal wash positive for anti-chlamydia antibody, whereas of the TC0439-challenged mice, 0 of 5 rectally infected and 1 of 5 infected by gavage were vaginal wash positive for anti-chlamydia antibody. Collectively, these results confirmed that TC0439 was highly attenuated for GI infection.

### GI attenuation of TC0439 is independent of challenge dose.

**FIG 6:**
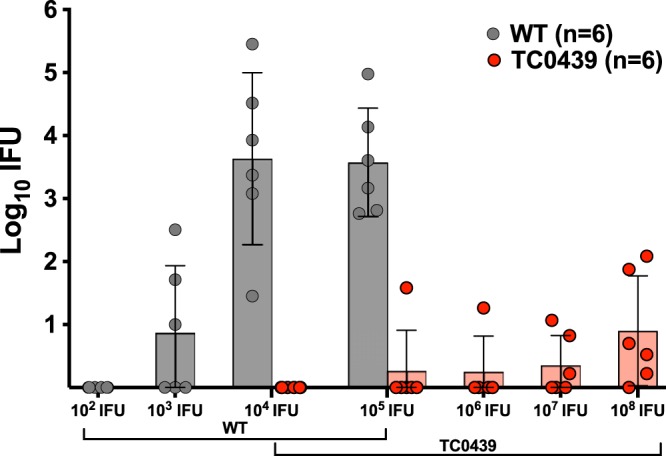
ID_50_ of TC0439 for GI infection is 1,000-fold greater than that of the WT. Mice were challenged intrarectally with the indicated doses of either the WT or mutant TC0439, and infection was monitored by swabbing the rectum and enumerating the IFUs on HeLa cell monolayers. Data are presented as the geometric mean ± SD at day 21 postchallenge. The calculated ID_50_ for the WT is 10^2.97^ (95% confidence interval, 2.23 to 3.65), and that for TC0439 is 10^6.85^ (95% confidence interval, 6.18 to 7.59); *P* < 0.0003.

GI infection established by either oral gavage or intrarectal inoculation of the WT resulted in similar infection courses, based upon rectal bacterial shedding and serology (Fig. 2 and 3). Therefore, we used the intrarectal inoculation model to compare the infectious dose responses of the WT and TC0439 (Fig. 6). For the purpose of calculating a 50% infectious dose (ID_50_), we evaluated infection at 21 days postchallenge. Based upon our previous experiments (Fig. 2), 21 days postchallenge was sufficient to establish, but not resolve, infection. The dose response to WT infection was assessed by challenging mice by intrarectal inoculation with either 10^2^, 10^3^, 10^4^, or 10^5^ IFUs. All mice challenged with 10^5^ or 10^4^ IFUs became infected, whereas intrarectal challenge with 10^3^ or 10^2^ IFUs resulted in GI infection of 3 of 6 and 0 of 6 mice, respectively. The estimated ID_50_ for WT GI infection by intrarectal inoculation was 10^2.97^ IFUs (95% confidence interval [CI], 10^2.23^ to 10^3.65^). Challenge doses of 10^4^, 10^5^, 10^6^, 10^7^, and 10^8^ IFUs were used to assess the dose response of TC0439. None of the doses were sufficient to infect 100% of the mice. Five of six mice inoculated with 10^8^ IFUs were infected, but the shedding of chlamydiae from these animals was much lower than that of animals infected with WT. The estimated ID_50_ for TC0439 GI infection by intrarectal inoculation was 10^6.85^ IFUs (95% CI, 10^6.18^ to 10^7.59^), which was significantly different from that of the WT (*P* < 0.0003). Not only was TC0439 more than a 1,000-fold less infectious than the WT for GI infection, but mice that did become culture positive following challenge with high doses of the mutant shed >100-fold fewer chlamydiae than mice infected with the WT. Ninety-four days following challenge, sera were analyzed for anti-chlamydial antibody. All culture-positive (infected) mice that had been inoculated with either the WT or mutant TC0439 were seropositive, whereas none of the culture-negative mice seroconverted, thus verifying the infectivity data.

### Background chromosomal nonsense mutation might explain profound GI attenuation.

**FIG 7:**
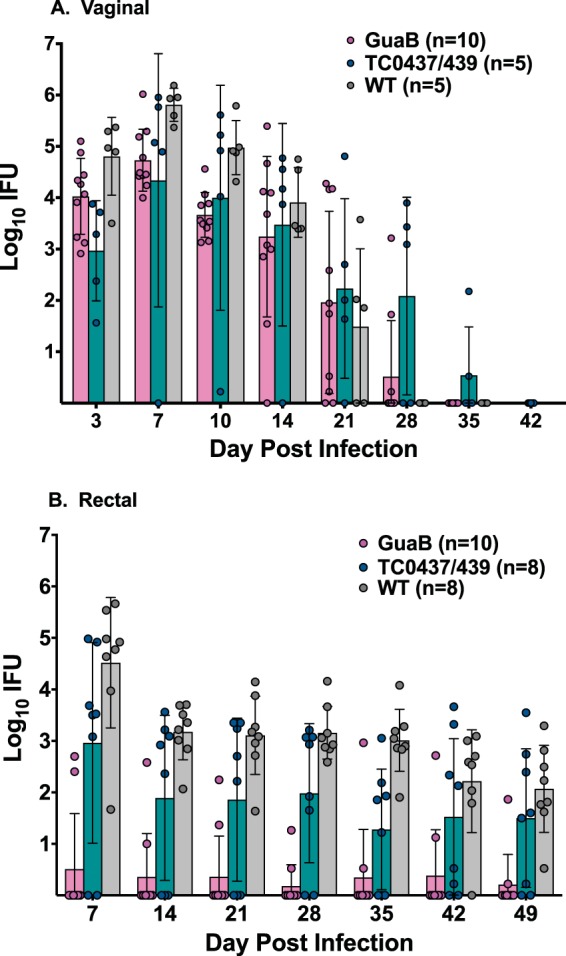
C. muridarum strains with nonsense mutations in *tc0600* are highly attenuated for GI infection. (A) Mice were pretreated with medroxyprogesterone acetate and challenged with 5 × 10^4^ IFUs of C. muridarum WT (*n* = 5), GuaB (*n* = 10), or TC0437/0439 (*n* = 5). Vaginal vaults were swabbed at the indicated times, and IFUs were enumerated on HeLa cell monolayers by immunofluorescence. Data are presented as the geometric mean ± SD of IFUs recovered at the indicated days postinfection. Both mutants displayed vaginal infections similar to that of the WT, although somewhat lower levels of chlamydial shedding of the mutants than of the WT were observed. The overall interaction effect between strains was not different (*P* > 0.22). However, results of tests comparing mutant GuaB and TC0437/0439 and WT main effects were statistically significant (*P* < 0.005). (B) Mice were inoculated intrarectally with 10 μl containing 1 × 10^5^ IFUs of either the WT (*n* = 8), GuaB (*n* = 10), or TC0437/0439 (*n* = 8). Infection was assessed weekly by swabbing the rectum and counting the IFUs on HeLa cell monolayers. Data are presented as the geometric mean ± SD of IFUs at the indicated time points. The overall interaction effect between the strains was not statistically significant (*P* > 0.11); however, results of tests comparing GuaB to WT, TC0437/0439 to WT, and GuaB to TC0437/0439 main effects were significant (*P* < 0.0001, *P* < 0.014, and *P* < 0.0006, respectively).

**FIG 8:**
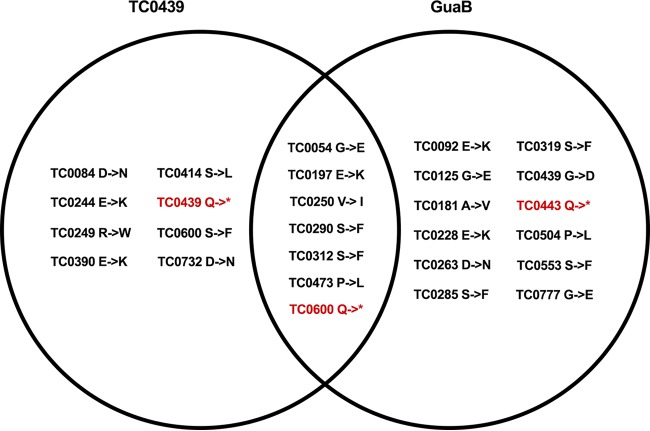
Venn diagram of the missense and nonsense mutations found in TC0439 and GuaB. An asterisk represents a stop codon.

TC0439 was generated by four sequential rounds of chemical mutagenesis and contains 28 point mutations in comparison to the WT, including nonsense mutations in *tc0439* and the conserved chlamydial open reading frame (ORF) *tc0600* (Table 1). To determine if GI attenuation of TC0439 might be linked to the inactivation of *tc0439*, *tc0600*, or other missense mutations, we evaluated the phenotypes of two additional mutants, TC0437/0439 and GuaB (Table 1; see Fig. 8), in the genital tract and GI infection models. TC0437/0439 contains nonsense mutations in *tc0437* and *tc0439*, while GuaB contains nonsense mutations in *guaB* and *tc0600*. Genital infection resulting from vaginal challenge with TC0437/0439 and GuaB resembled WT infection (Fig. 7A), although chlamydial shedding was somewhat reduced compared to that of the WT (*P* < 0.05). However, when the mutants were compared to the WT in the rectal infection model, TC0437/0439 behaved similarly to the WT, though shedding was somewhat reduced (*P* < 0.05), whereas the mutant GuaB was strikingly attenuated (*P* < 0.00001), with few animals displaying evidence of productive infection (Fig. 7B). The marked attenuation of TC0439 and GuaB for GI infection provides compelling evidence that distinct chromosomal genes strongly impact the tissue-specific virulence of Chlamydia. Furthermore, a shared mutation in the genomes of TC0439 and GuaB (Table 2; Fig. 8) resulted in dramatic GI attenuation.

**TABLE 1:** Summary of SNPs in C. muridarum mutants

Mutant strain	Total no. of mutations	No. of silent mutations	No. of missense mutations	No. of intergenic mutations	Nonsense mutation characteristics			
					Gene	Genomic position	Nucleotide change	Amino acid change
TC0437	44	13	29	0	*tc0412*	473585	C→T	Q→STOP
					*tc0437*	506777	C→T	Q→STOP
TC0438	35	6	26	0	*tc0412*	473585	C→T	Q→STOP
					*tc0438*	516403	C→T	Q→STOP
					*tc0450*	549083	C→T	R→STOP
TC0439	28	10	13	3	*tc0439*	527311	C→T	Q→STOP
					*tc0600*	717761	G→A	Q→STOP
TC0437/0439	53	15	35	0	*tc0412*	473585	C→T	Q→STOP
					*tc0437*	506777	C→T	Q→STOP
					*tc0439*	526979	G→A	W→STOP
GuaB	29	8	17	2	*tc0443*	541698	G→A	Q→STOP
					*tc0600*	717761	G→A	Q→STOP

**TABLE 2:** Mutations present in both TC0439 and GuaB mutant strains

Genomic position	Gene ID	Description	Nucleotide change	Amino acid change
62823	*tc0054*	Penicillin binding protein	G→A	Gly→Glu
126188	Intergenic between *tc0106* and *tc0107*	Intergenic	C→T	Intergenic
224779	*tc0191*	Hypothetical protein	G→A	Gly→Gly
233168	*tc0197*	Polymorphic membrane protein, PmpD	G→A	Glu→Lys
292616	*tc0250*	Hypothetical protein	C→T	Val→Ile
349120	*tc0290*	Hypothetical protein	G→A	Ser→Phe
369812	*tc0312*	Glycosyl hydrolase, GlgX	G→A	Ser→Phe
573799	*tc0473*	Peptide ABC transporter, permease protein	C→T	Pro→Leu
717761	*tc0600*	Hypothetical protein	G→A	Gln→STOP

## DISCUSSION

Chlamydia spp. colonize multiple distinct organ system tissues (7). In humans, C. trachomatis strains most frequently infect the cervix and urethra but can also colonize the pharynx, conjunctiva, and GI tract. Chlamydial GI infections are often asymptomatic, can persist without overt signs or symptoms of infection, and are less sensitive to the standard-of-care antibiotic therapy (single-dose azithromycin) (6, 8, 9, 26–28). Because of the propensity of GI infections to persist, even following antibiotic therapy, it has been proposed that chlamydial GI infection is a reservoir for genital tract reinfections (7–10). The recent realization that chlamydia GI infections are common in women has renewed interest in understanding the pathogenesis and the pathological consequences of GI chlamydial infection and in elucidating the role of these infections in the epidemiology of the more problematic female urogenital infections.

The murine model using C. muridarum is an ideal experimental system to study tissue-specific virulence factors of Chlamydia. C. muridarum colonizes both the genital and GI tracts and produces distinct tissue-specific experimental outcomes that mimic many characteristics of human genital and GI infections. GI colonization with C. muridarum can be established by oral or rectal inoculation or as the result of dissemination via the blood from a genital infection (29). GI infection often persists and is characterized by long-term shedding of infectious chlamydiae in the absence of overt inflammation (10). Conversely, genital infection is characterized by the shedding of abundant infectious chlamydiae and significant inflammation, and infection naturally resolves in about 4 weeks (30, 31).

A few studies have used the murine infection model to study chlamydial tissue tropism (32–34). In one recent study, the C. muridarum plasmid gene *pgp3* was associated with GI but not genital infection (34). However, our current study clearly demonstrates that tissue-specific virulence factors are also encoded on the C. muridarum chromosome. We speculate that a nonsense mutation in the chromosomal gene *tc0600* was independently associated with GI-specific attenuation, but further genetic studies are needed to precisely map the chromosomal gene(s) responsible for the GI-specific attenuation. Interestingly, polymorphisms in the C. trachomatis *tc0600* ortholog, *ct326*, which encodes a putative secreted inclusion membrane protein (35), have been associated with GI tropism in humans. A 111-nucleotide in-frame insertion in *ct326* of C. trachomatis serovar G is correlated with the ability of this strain to infect the GI tract, and strains lacking this insertion are associated with cervical but not GI infection (36). This 111-nucleotide region is conserved in *C. muridarum tc0600*.

Although we associated inactivation of *tc0600* with GI attenuation, the TC0439 and GuaB mutant strains share seven additional nonsynonymous mutations (Table 2; Fig. 8). The shared mutations suggest that these isolates were derived from a common parent that was present in the population after the first few rounds of mutagenesis. Four of these mutations are predicted to cause nonconserved amino acid changes in TC0054 (penicillin binding protein), TC0290 (hypothetical), TC0312 (glycosyl hydrolase, GlgX), and TC0473 (peptide ABC transporter, permease protein). These genes have not been linked to C. muridarum virulence or reported to be plasmid regulated (37–39). Identifying the specific allele(s) responsible for attenuation of the TC0439 and GuaB mutant strains is essential to understanding the mechanism of GI attenuation. Unfortunately, complementation is contraindicated for differentiating the potential mutant alleles, because the plasmid is a C. muridarum GI virulence factor (32) and negative complementation results could indicate a failure to complement or *cis*-acting effects on the plasmid. Perhaps one method that could be used to circumvent this concern is the fluorescence-reported allelic exchange mutagenesis (FRAEM) approach developed in C. trachomatis to inactivate *tc0600*, which would permit generation of targeted chromosomal deletions while maintaining the endogenous plasmid (40).

Also of interest is the nonsynonymous single-nucleotide polymorphism (SNP) in *tc0439* contained in the GuaB mutant (amino acid [aa] residue 3114 of the TC0439 mutant), which results in a glycine-to-aspartic acid change. Sequence comparison of cytotoxin genes from a variety of pathogens shows that this glycine residue is highly conserved, and thus mutating such a highly conserved residue could result in the loss of toxin function. Because this *tc0439* mutation is found in the GuaB mutant and the TC0439 mutant contains a premature stop codon in *tc0439*, perhaps the attenuated GI infection phenotype observed with these two mutants was due to loss of function of the *tc0439* toxin. However, the TC0437/0439 mutant, which contains a premature stop codon in *tc0439* upstream of the nonsynonymous SNP in *tc0439* contained in the GuaB mutant, remains infectious for the GI tract and thus does not corroborate that notion. The multiple mutations present in our strains confound the identification of a specific gene mutation responsible for the tissue-tropic phenotype with absolute certainty, and the infection phenotype that we report could be multifactorial. Nevertheless, the very robust *in vivo* infection phenotype provides a compelling basis for further studies to elucidate the molecular mechanism of tissue-specific infection.

Short-lived protective immunity develops following human C. trachomatis genital infection (41–43), whereas in mice, protective immunity is quite long-lasting (30, 44, 45). This dissimilarity in the duration of immunity between humans and mice is not understood and could be multifactorial. However, our observations and those of others showing that murine GI C. muridarum infection produces nearly sterilizing immunity against vaginal challenge (Fig. 4) (23) may be central to elucidating the immunological mechanisms responsible for the durability of chlamydial immunity. Mice infected vaginally with C. muridarum concurrently acquire GI infection and remain GI tract positive long after genital infection has resolved (46). Perhaps, then, persisting GI infection is a vital component for the robust durability of protective immunity and could be key to developing an efficacious vaccine to prevent chlamydial genital infection. We clearly showed that genital and GI infection tropisms were separable by identifying a mutant that infected the genital tract normally but was highly attenuated for GI infection. While that is not the phenotype desired for a chlamydial vaccine, it does provide proof of principle for the identification of strains that possess altered tissue tropism. Identification of strains that infect the GI tract but are attenuated for genital tract infection would allow for direct testing of the hypothesis.

By using animal models of infection, a more complete understanding of the contribution of chlamydial GI infection to the robust and long-lasting protective immunity that develops against genital infection will emerge. However, well-designed epidemiological studies and correlative immunological analysis of subjects with chlamydial urogenital and GI infections are needed to determine if a link exists between GI infection and protective immunity to genital chlamydia infection in humans.

## MATERIALS AND METHODS

### Chlamydia strains.

C. muridarum wild-type (WT) (GenBank accession AE002160.2) and C. muridarum mutants TC0437, TC0438, TC0439, GuaB (17), and TC0437/0439 (see Table S1 in the supplemental material) were propagated in HeLa 229 (47) cells, and elementary bodies (EBs) were purified by discontinuous Renografin gradient centrifugation. Table 1 summarizes the single-nucleotide polymorphisms (SNPs) found in the mutants (17) (Table S1).

### Generation and whole-genome sequencing of C. muridarum TC0437/0439.

TC0437 (Table 1) was mutagenized with 8 mg/ml ethyl methanesulfonate (EMS), and a clone that contained a nonsense mutation in *tc0439* (TC0437/0439) was identified, purified, and sequenced as described previously (17). SNPs and nucleotide insertions and deletions (indels) were mapped by aligning the TC0437/0439 and C. muridarum reference genomes (GenBank accession number AE002160.2) using Bowtie2. SNPs/indels were called using a Samtools mpileup function, and the remaining ambiguous sequences with low-quality scores were resolved by Sanger sequencing.

### Mice.

Female C57BL/6 mice, 6 to 8 weeks old, were purchased from Jackson Laboratories (Bar Harbor, ME) and maintained in the animal facilities at the University of Arkansas for Medical Sciences (Little Rock, AR). All experimental procedures were performed in accordance with protocols approved by the UAMS Institutional Animal Care and Use Committee.

### Genital infection.

Five days before genital infection, mice were injected subcutaneously with 2.5 mg of medroxyprogesterone acetate (Depo-Provera; Greenstone, LLC) to synchronize the estrous cycle of experimental mice. Mice were then infected vaginally with 5 × 10^4^ IFUs of the WT or mutants (48, 49). Infections were monitored by inoculating cervicovaginal swab samples onto HeLa 229 cell monolayers, and IFUs were visualized and counted as described in detail previously (48, 49).

### GI infection.

Two routes of inoculation, intrarectal and gavage, were used to assess the pathogenicity of the mutants for the murine gastrointestinal tract. For rectal inoculation, the tip of a micropipette was gently inserted approximately 5 mm into the rectum, and 10 μl of a buffered suspension (250 mM sucrose, 10 mM sodium phosphate, and 5 mM l-glutamic acid, pH 7.2) (SPG) containing 1 × 10^7^ IFUs/ml of WT or mutants (1 × 10^5^ IFU, total challenge dose) was deposited. Infection was monitored by collecting rectal swabs at weekly intervals. Calgiswabs (Puritan) were wetted with SPG, inserted into the rectum, and rotated 8 times clockwise and 8 times counterclockwise. Swabs were then placed in a tube containing 0.5 ml SPG and two 4-mm glass beads and vortexed at 1,400 rpm for 3 min. The swabs were removed, an additional 0.5 ml of SPG was added, and samples were stored at −80°C. Infection was monitored by counting IFUs as described previously (48).

A flexible 20-gauge feeding tube (Instech) was used to challenge mice by gavage. Mice were inoculated with 1 × 10^6^ IFUs of the WT or mutants by depositing 100 μl of a suspension of 1 × 10^7^ IFUs/ml in SPG into the stomach. Gastrointestinal infection was monitored by rectal swabs, and IFUs were counted as described above (48).

### Vaginal challenge of GI infected mice.

Mice were infected with either the WT or TC0439 by rectal or gavage inoculation, and infection was monitored weekly. One hundred eighteen days following GI infection, mice received daily intraperitoneal injections of 300 μg of doxycycline for 10 days, which is sufficient to cure GI infection (9). The mice were then rested for 2 weeks to eliminate any residual effect of the antibiotic treatment, and rectal swabs confirmed that the mice were culture negative for chlamydiae. The mice were then treated with medroxyprogesterone acetate and challenged vaginally with the homologous strain (WT or TC0439) as described above for genital infection.

### ELISA.

Anti-chlamydial antibody was measured by enzyme-linked immunosorbent assay (ELISA) using formalin-fixed, density gradient-purified WT EBs as the antigen (30, 49).

### Statistical analyses.

Analyses of bacterial shedding data were performed using nonparametric longitudinal analysis methods (50). The methods are rank based and require fewer distributional assumptions than parametric methods. The models included terms representing group (chlamydial strain), time (day of measurement), and a group-by-time interaction. As with other factorial models, the interaction effect was evaluated first, and, if not significant, the overall group and time effects could be assessed. However, in the case of a significant interaction, the time profiles of each mutant were compared to those of the WT, separately. Mutants whose time profile differed from that of the WT were examined further by comparing the mutant to the WT at each time point. Wilcoxon rank sum tests were used for these analyses. Wilcoxon rank sum tests were also used to compare mutants to WT with respect to antibody titer data. Finally, probit regression models were used to obtain ID_50_ estimates and corresponding 95% confidence limits. *P* values of <0.05 were considered to be statistically significant. No adjustment for multiple comparisons was made.

## Supplementary Material

Supplemental material
