# Supplementary material for: Chlamydia muridarum Genital and Gastrointestinal Infection Tropism Is Mediated by Distinct Chromosomal Factors
Source: Infect Immun. 2018 Jun 21;86(7):e00141-18. doi: 10.1128/IAI.00141-18 (PMC6013670; doi:10.1128/IAI.00141-18)
Supplement: Supplemental material [file supp_86_7_e00141-18__index.html]

Supplemental material 

# Chlamydia muridarum Genital and Gastrointestinal Infection Tropism Is Mediated by Distinct Chromosomal Factors

## Supplemental material

- Supplemental file 1 -

  Table S1. Single nucleotide polymorphisms.

  PDF, 51K
