# Supplementary material for: Chlamydia muridarum Genital and Gastrointestinal Infection Tropism Is Mediated by Distinct Chromosomal Factors
Source: Infect Immun. 2018 Jun 21;86(7):e00141-18. doi: 10.1128/IAI.00141-18 (PMC6013670; doi:10.1128/IAI.00141-18)
Supplement: Supplemental material [file IAI.00141-18_zii999092442s1.pdf]

Table S1. Single nucleotide polymorphisms identified in the TC0437/TC0439 double mutant in comparison to *C. muridarum* nigg as described in Rajaram et al. 2015

TC0437/TC0439

Gene ID	Description	Nucleotide Position	Nucleotide Change	Amino Acid change
TC0008	exodeoxyribonuclease V, gamma subunit, putative	13703	G -> A	T -> I
TC0019	recA	26275	G -> A	E -> K
TC0023	ABC transporter, ATP-binding protein	30512	G -> A	Y -> Y
TC0035	conserved hypothetical protein	40681	G -> A	L -> L
TC0069	endonuclease III	80096	C -> T	P -> S
TC0074	preprotein translocase SecA subunit, secA	85403	C -> T	R -> C
TC0096	ribosomal large subunit pseudouridine synthase B, rluB	115068	G -> A	G -> G
TC0104	3,4-dihydroxy-2-butanone-4-phosphate synthase/GTP cyclohydrolase II	123697	G -> A	V -> I
TC0120	conserved hypothetical protein	144193	C -> T	R -> C
TC0125	alanyl-tRNA synthetase	153963	G -> A	A -> V
TC0137	UDP-N-acetylmuramoylalanyl-D-glutamyl-2,6-diaminopimelate--D-alanyl-D-alanyl ligase MurF, putative	168334	C -> T	L -> L
TC0159	primosomal protein N	193263	G -> A	R -> R
TC0172	conserved hypothetical protein	202909	G -> A	G -> E
TC0213	CDP-diacylglycerol--serine O-phosphatidyltransferase, putative	252676	C -> T	M -> I
TC0218	UDP-N-acetylenolpyruvoylglucosamine reductase, murB	260165	G -> A	S -> F
TC0229	cell division protein; FtsH	271271	C -> T	L -> F
TC0237	conserved hypothetical protein	277523	G -> A	R -> C
TC0263	polymorphic membrane protein; pmpG-1	311751	C -> T	P -> S
TC0283	PhoH-related protein	338843	C -> T	G -> R
TC0290	conserved hypothetical protein	349249	C -> T	R -> K
TC0330	protein export protein, FHIPEP family protein	391857	C -> T	R -> W
TC0370	ribosome-binding factor A, rbfA	430750	C -> T	G -> E

TC0383	A/G-specific adenine glycosylase	444982	A -> G	T -> A
TC0412	conserved hypothetical protein	473585	C -> T	Q -> STP
TC0424	conserved hypothetical protein	487908	C -> T	A -> V
TC0437	adherence factor	506777	C -> T	Q -> STP
TC0438	adherence factor	520769	G -> A	R -> Q
TC0439	adherence factor	526979	G -> A	W -> STP
TC0453	conserved hypothetical protein	551457	G -> A	R -> R
TC0460	thymidylate kinase	558168	C -> T	E -> K
TC0469	conserved hypothetical protein	569879	C -> T	A -> V
TC0471	peptide ABC transporter, periplasmic peptide-binding protein, putative	571980	C -> T	A -> V
TC0471	peptide ABC transporter, periplasmic peptide-binding protein, putative	572022	C -> T	T -> I
TC0479	pyrophosphate--fructose 6-phosphate 1-phosphotransferase, beta subunit, pfkA-2	581345	G -> A	E -> K
TC0490	Rep helicase family protein; uvrD	596078	G -> A	S -> S
TC0501	sodium:dicarboxylate symporter family protein	607577	C -> T	S -> F
TC0544	conserved hypothetical protein	654353	G -> A	I -> I
TC0575	serine/threonine kinase protein	684627	G -> A	A -> A
TC0588	DNA-directed RNA polymerase, beta subunit, rpoC	702107	G -> A	L -> F
TC0602	helicase, putative	721368	C -> T	R -> K
TC0635	conserved hypothetical protein	761471	C -> T	G -> E
TC0645	3-phosphoshikimate 1-carboxyvinyltransferase, aroA	772383	G -> A	K -> K
TC0694	polymorphic membrane protein B/C family protein; pmpB/C-1	829608	C -> T	N -> N
TC0694	polymorphic membrane protein B/C family protein; pmpB/C-1	831869	C -> T	S -> F
TC0711	conserved hypothetical protein	850235	G -> A	G -> R
TC0733	secDF protein, putative	872206	G -> A	D -> D
TC0810	ribosomal protein, L22, rplV	945533	C -> T	G -> R
TC0816	conserved hypothetical protein	949915	C -> T	T -> T
TC0833	uracil phosphoribosyltransferase, upp	969350	G -> A	G -> R
TC0842	branched-chain amino acid transport system carrier protein, putative	977030	G -> A	G -> E

TC0864	DNA mismatch repair protein MutL	1001277	G -> A	L -> F
TC0877	regulatory protein, putative	1016507	C -> T	H -> Y
TC0917	geranylgeranyl pyrophosphate synthase, putative	1069215	G -> A	L -> L

Grey highlighted: Indicates mutations that differ from the tc0437 parent. The TC0437/TC0439 mutant was generated from the TC0437 mutant by subsequent rounds of EMS mutagenesis (see materials and methods).
